# Integrating a Large Language Model to Streamline Nursing Handover Documentation Across Multiple Hospitals in Taiwan: Development and Implementation Study

**DOI:** 10.2196/81604

**Published:** 2026-03-12

**Authors:** Ray-Jade Chen, Mai-Szu Wu, Lung-Wen Tsai, Shy-Shin Chang, Shu-Tai Shen Hsiao, Yu-Sheng Lo

**Affiliations:** 1 Department of Surgery School of Medicine, College of Medicine Taipei Medical University Taipei Taiwan; 2 College of Medicine Professional Master Program in Artificial Intelligence in Medicine Taipei Medical University Taipei Taiwan; 3 Department of Surgery Division of Pediatric Surgery Taipei Medical University Hospital Taipei Taiwan; 4 Department of Internal Medicine, School of Medicine College of Medicine Taipei Medical University Taipei Taiwan; 5 Division of Nephrology Department of Internal Medicine Shuang Ho Hospital New Taipei City Taiwan; 6 Taipei Medical University Research Center of Urology and Kidney (TMU-RCUK) Taipei Medical University Taipei Taiwan; 7 Graduate Institute of Data Science College of Management Taipei Medical University New Taipei City Taiwan; 8 Department of Information Technology Office Taipei Medical University Hospital Taipei Taiwan; 9 Department of Medicine Research Taipei Medical University Hospital Taipei Taiwan; 10 Department of Family Medicine School of Medicine, College of Medicine Taipei Medical University Taipei Taiwan; 11 Department of Family Medicine Taipei Medical University Hospital Taipei Taiwan; 12 Office of Superintendent Taipei Medical University Hospital Taipei Taiwan; 13 School of Nursing Taipei Medical University Taipei Taiwan; 14 Graduate Institute of Biomedical Informatics College of Medical Science and Technology Taipei Medical University New Taipei City Taiwan; 15 Center of Digital Innovation and Development Taipei Medical University New Taipei City Taiwan

**Keywords:** nursing shortage, large language model, generative artificial intelligence, artificial intelligence, nursing information system, electronic medical records

## Abstract

**Background:**

The global nursing shortage, exacerbated by heavy workloads and high turnover rates associated with the COVID-19 pandemic, continues to undermine care quality and nurse well-being. Although digital health technologies have enhanced coordination, improved communication, and reduced clinical errors in nursing practice, they have also increased nurses’ documentation burden. Advances in large language models (LLMs) and other generative artificial intelligence (GenAI) tools facilitate the generation of accurate reports from electronic medical records (EMRs), thereby streamlining documentation workflows, saving time, and reducing nurses’ workloads. Accordingly, integrating LLMs into electronic nursing documentation systems warrants further exploration.

**Objective:**

This study examines the integration of an LLM into an in-house nursing information system (NIS) implemented across 3 hospitals in Taiwan to reduce the time and effort required for nursing handover documentation and to preliminarily assess the operational and economic implications of GenAI-assisted workflows.

**Methods:**

A multidisciplinary team of nursing specialists and information technology experts at Taipei Medical University (TMU) restructured the organization’s existing nursing handover documentation process to facilitate interaction with the LLM. The team also developed prompt-based interfaces to automatically generate section-specific content for the nursing handover document. The LLM-integrated NIS was subsequently deployed across 3 hospitals in Taiwan: Taipei Medical University Hospital (TMUH), Wan Fang Hospital (WFH), and Shuang Ho Hospital (SHH). We then extracted and analyzed NIS log data to compare documentation times before and after LLM implementation, thereby quantifying time savings.

**Results:**

Integration of the LLM into nursing handover documentation was associated with shorter per-patient documentation time in routine clinical use across TMUH, WFH, and SHH. Based on preintegration NIS logs (September 2024), the average handover document completion time per patient ranged from 3.45 (SD 3.82) to 4.32 (SD 4.48) minutes across hospitals and shifts, providing a preliminary baseline for subsequent comparisons. In postintegration NIS logs (October-December 2024), the overall handover document completion time per patient (mean) was substantially lower, ranging from 1.17 (SD 1.86) to 2.54 (SD 2.82) minutes across hospitals and shifts. Using monthly patient volume to estimate time savings, 113-273, 160-314, and 198-391 hours were saved per month at TMUH, WFH, and SHH, respectively, corresponding to aggregate savings of 474-981 hours per month across hospitals during the study period.

**Conclusions:**

We integrated an LLM into an NIS to generate nursing handover documents without altering existing workflows. Across 3 hospitals within TMU’s health system, GenAI assistance was associated with shorter documentation time and a positive net labor value from October to December 2024. Prompts were constrained, and nurse verification was required to mitigate hallucinations. Future work will enhance logging to capture reliability and editing metrics, compare LLM-generated drafts with nurse-finalized notes to inform prompt refinement, and assess generalizability to other documentation workflows.

## Introduction

The global nursing shortage has been exacerbated by several factors and remains a critical challenge for health care systems, threatening health care quality and access [1,2]. The COVID-19 pandemic has increased nursing workloads, resulting in increased patient acuity, greater infection control demands, and longer working hours—all of which have contributed to nurses experiencing physical exhaustion, moral distress, and mental health challenges [3,4]. Moreover, the pandemic worsened the nursing shortage, with 100,000 nurses leaving the US health care workforce during the pandemic and an additional 900,000 planning to exit by 2027 [5]. This shortage has compromised care quality and increased burnout and turnover risks. According to the World Health Organization (WHO), although the global nursing workforce grew from 27.9 million in 2018 to 29.8 million in 2023, a shortage of 5.8 million nurses persists, which is projected to decrease to 4.1 million by 2030 [6].

Although Taiwan experienced early success in controlling the COVID-19 pandemic, the pandemic exposed structural problems within the country’s nursing sector. Nursing shortages, particularly in intensive care and emergency departments, persisted during the late-pandemic and postpandemic periods. High turnover rates and uneven workloads contributed to increased work-related stress [7]. Furthermore, interviews with public health nurses revealed that frequent changes in temporary assignments and information systems during the pandemic exacerbated fatigue and created additional challenges for frontline nurses [8].

The ongoing digital transformation in health care has rendered digital technologies essential to nursing practice. Studies have indicated that the adoption of digital tools by health care professionals improves care quality, enhances patient participation in their own care [9,10], fosters better coordination among health care professionals [11], reduces medication errors and adverse events [12,13], and improves documentation accuracy [14,15]. However, this shift has led to more time being spent on indirect tasks, particularly activities related to maintaining electronic medical records (EMRs), including data collection, entry, and confirmation [16]. Documentation accounts for a considerable portion of nurses’ work time, and this burden is exacerbated by high patient turnover [17] and the use of multiple recording systems [18,19]. A systematic review revealed that nurses spend between 13% and 31% of their work time on documentation [20]; thus, considering nurses’ heavy workloads, reducing documentation time has become a critical issue in nursing.

Large language models (LLMs), a category of artificial intelligence (AI) tools, perform deep learning with millions or billions of parameters on extensive data to generate human-like text [21]. LLM platforms, such as ChatGPT (OpenAI), Gemini (Google LLC), and Llama (Meta Platforms, Inc.) [22,23], are applied in various domains, including text generation, question answering, translation, and creative content creation. Moreover, large multimodal models have been developed to process several types of data, such as text, images, video, and audio, thereby broadening the application potential of generative AI (GenAI) tools [24,25]. In health care, LLMs and other GenAI tools enable coherent text generation for tasks such as drug discovery, personalized medicine, radiology diagnosis, automated clinical documentation, medical research, and health information consultation [26-34]. These tools are driving advancements in diverse health care applications; however, ensuring the accuracy, reliability, and fairness of the generated results remains critical [35].

AI applications in nursing are rapidly expanding, and AI tools have the potential to become essential parts of nursing practice [36,37]. By integrating clinical data with language generation tools, GenAI systems can improve documentation quality, support clinical decision-making, and reduce administrative workload. Additionally, these systems can considerably shorten documentation time and enhance handover efficiency by rapidly producing consistent records, thereby allowing nurses to devote more time to patient care and ultimately improving the quality and safety of health care delivery [38,39].

To explore the potential of incorporating LLMs into electronic nursing documentation systems, this study integrated an LLM into an in-house nursing information system (NIS) to reduce the time and effort required for nursing handover documentation. We evaluated the LLM-integrated NIS by comparing the time spent by nurses on nursing handover documentation before and after integration. We additionally conducted a preliminary cost-effectiveness assessment and quantified the revision rate of GenAI-generated drafts before final submission.

## Methods

### Settings

This study was conducted at Taipei Medical University (TMU), which operates 3 hospitals: Taipei Medical University Hospital (TMUH), Wan Fang Hospital (WFH), and Shuang Ho Hospital (SHH). The combined capacity of these hospitals is 2675 acute beds (TMUH: 735; WFH: 761; SHH: 1179), and they handle a total of over 400,000 outpatient visits per month. Owing to nursing shortages, operational capacity decreased to 2195 acute beds as of May 2025 (TMUH: 608; WFH: 576; SHH: 1011). Nursing workflows are supported by an in-house NIS managed by the TMU Center of Digital Innovation and Development.

### Google Gemini

We selected a HIPAA (Health Insurance Portability and Accountability Act)-compliant enterprise instance of Gemini 1.5 Pro (Google LLC) for integration into the in-house NIS. Gemini 1.5 Pro is a mid-sized multimodal LLM optimized for a broad range of reasoning tasks and can generate text output in response to diverse inputs, including text, image, video, and audio. Moreover, it offers capabilities for output streaming, conversational interactions, and system-level instruction handling. The model can be used to conduct various language-related tasks, such as text generation, text editing, code generation, problem-solving, recommendation generation, information extraction, and data generation [40].

### Conventional Workflow in Nursing Handover Documentation

In hospitals across Taiwan, nursing schedules are typically structured around 3 daily shifts, and nurses are required to complete handover documentation twice per day. The nursing handover document follows the WHO-endorsed ISBAR (Identify, Situation, Background, Assessment, Recommendation) framework structured into 5 key components [41].

**Figure 1:**
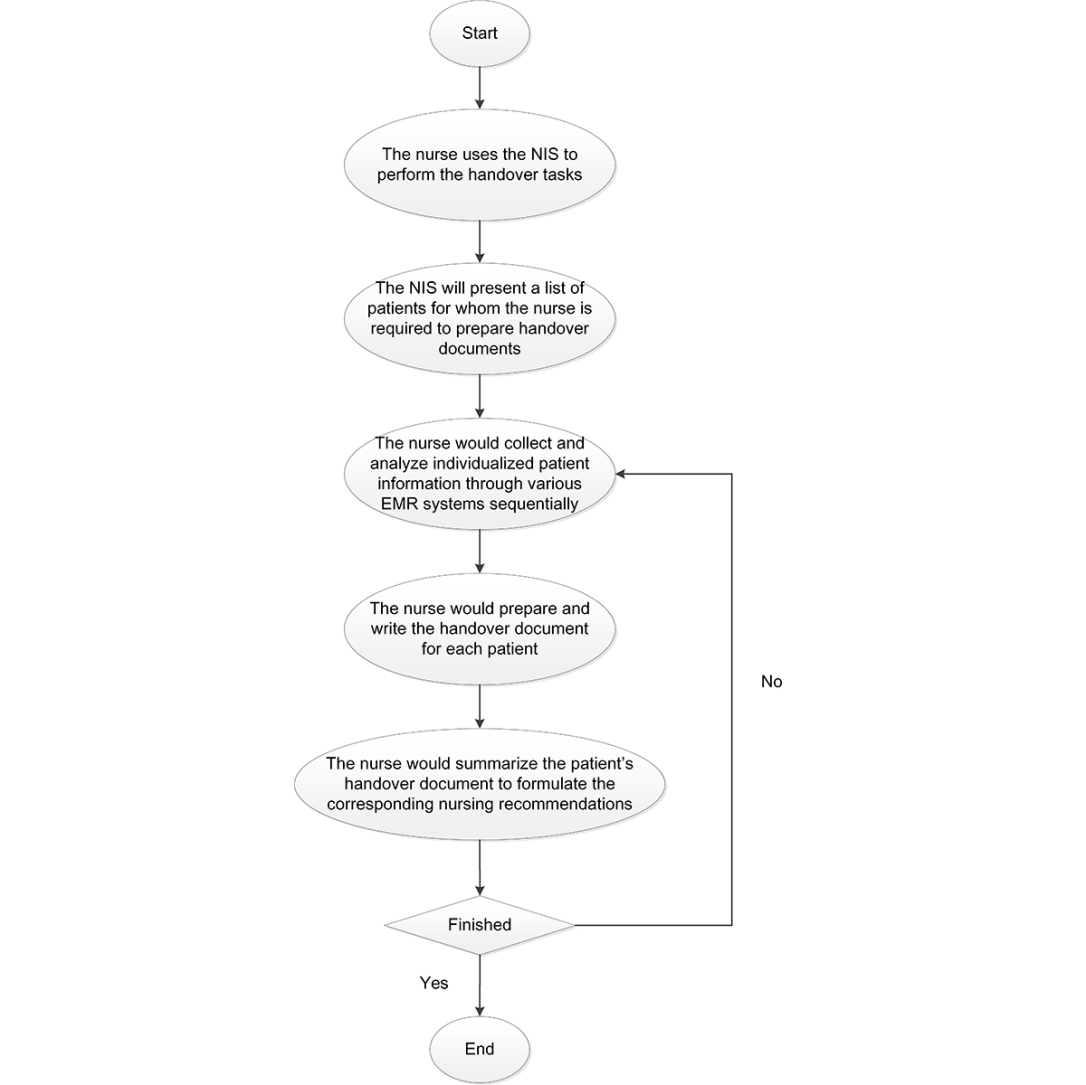
Conventional workflow for nursing handover documentation using the in-house nursing information system (NIS) at Taipei Medical University. EMR: electronic medical record.

Figure 1 illustrates the conventional workflow for nursing handover documentation with the in-house NIS. Initially, the NIS displays a list of patients requiring handover documentation. Subsequently, nurses collect and analyze individualized patient information using various EMR systems. They then sequentially write a nursing handover document for each patient. Finally, the documented handover information is manually summarized to formulate the corresponding nursing recommendations for each patient.

In general, nurses prepare handover documents for 5-7 patients per shift, and senior nurses estimated an average completion time of approximately 5 minutes per patient. Accordingly, each nurse dedicates approximately 1 hour to complete patient handover tasks; this time frame includes both the documentation process and the subsequent verification and explanation of patient details to the receiving nurse.

### Prompt Design for Nursing Handover Documentation

A multidisciplinary team of nursing specialists and information technology professionals collaboratively outlined the key components of the nursing handover document, as defined in the existing EMR systems. Each component was divided into several sections, which were systematically evaluated to assess the extracted content and identify potential problems. The most critical problem identified was related to the manual collection, verification, transcription, and integration of patient data from various EMR systems. Thus, we reorganized the structure of the nursing handover document. Tables 1 and 2, respectively, present the sections of the original and revised (for the LLM-integrated NIS) nursing handover documents across 5 key components according to the ISBAR framework.

We developed section-specific prompts to support nurses’ use of the LLM, targeting 4 tasks: (1) extracting information from the provided input, (2) summarizing input text, (3) interpreting or translating text (eg, English to Mandarin), and (4) formatting content based on the input. When executed, these prompts generate draft content for the corresponding handover sections. Prompt development followed an iterative, human-in-the-loop process: draft prompts were repeatedly tested using deidentified EMRs, and outputs were reviewed by nursing professionals for clinical relevance, completeness, and adherence to the intended structure. Feedback-informed refinements were made to prompt wording, section constraints, and output formatting until the drafts were suitable for routine handover use. All prompts instructed the LLM to use only the provided input and to avoid introducing new values, assumptions, or inferences. No patient identifiers or personally identifiable information was transmitted to the LLM.

For example, in the laboratory results section, the NIS queried the laboratory information system to retrieve each patient’s test results and corresponding reference ranges from the preceding 48 hours. A tailored prompt was then sent via the LLM application programming interface (API) to identify abnormal results by comparing values with reference ranges and to present the extracted abnormalities in a structured table. Table 3 summarizes the tailored prompts by section and the corresponding EMR source systems, including the inpatient system, medical documentation system, NIS, laboratory information system, examination information system, radiology information system, patient schedule system, operating room system, and blood bank system.

**Table 1:** The original nursing handover document.

Components	Sections
Identity	Introduction
Situation	Vital signs, blood glucose, laboratory test results, culture results, diagnostic examination reports, radiology reports, pending diagnostic examination tests or radiology tests, surgical records, and blood products
Background	Admission reason, medical history
Assessment	Wound records, consultation records
Recommendations	A blank field for manual data entry
Others	A blank field for manual data entry

**Table 2:** The revised (for the large language model–integrated nursing information system) nursing handover document.

Components	Sections
Identity	Introduction
Handover records^a^	Vital signs, blood glucose, laboratory test results, culture results, diagnostic examination reports, radiology reports, pending diagnostic examination tests or radiology tests, surgical records, blood products, wound records, consultation records, and recommendations
Background	Admission reason, medical history
Others	A blank field for manual data entry

^a^Handover records include records related to the situation (vital signs, blood glucose, laboratory test results, culture results, diagnostic examination reports, radiology reports, pending diagnostic examination tests or radiology tests, surgical records, and blood products), assessment (wound records and consultation records), and recommendations.

**Table 3:** Tailored prompts by handover section and corresponding electronic medical record source systems.

Components and sections			Electronic medical record source systems	Tailored prompts
**Identity**				
	Introduction	Inpatient system		The nursing information system already provides information (manual data entry is not necessary).
**Background**				
	Admission reason and medical history	Inpatient system and medical documentation system		Summarize admission details into a concise paragraph. Highlight the primary reason for admission and include key clinical details.
**Handover records**				
	Vital signs	Nursing information system		Extract and structure the following vital signs data from the past 8 hours from the provided data: date, time, temperature, pulse, respiration, blood pressure, oxygen level, and Glasgow Coma Scale values. Maintain chronological order.
	Blood glucose	Laboratory information system		Extract and structure blood glucose readings from the past 8 hours, including date, time, and measurement value. Organize the data in reverse chronological order.
	Laboratory test results	Laboratory information system		Extract the following laboratory results from the past 48 hours: test item, value, and reference range. Mark abnormal values (H for high and L for low), and leave normal values unmarked. Organize the results chronologically and return them in a structured JSON format.
	Culture results	Laboratory information system		Extract and structure key information from culture data within 6 days. For each data sample, identify and output the following fields in a structured format: collection date, specimen type, culture results, and status (completed or pending).
	Diagnostic examination reports	Examination information system		Summarize diagnostic findings from the past 24 hours, emphasizing significant abnormal results in Standard Mandarin. If such findings are unavailable, state “No examination reports available.”
	Radiology reports	Radiology information system		Summarize key radiological findings from the past 24 hours, highlighting clinically relevant abnormalities in Standard Mandarin. If such findings are unavailable, state “No radiology reports available.”
	Pending diagnostic examination tests or radiology tests	Patient schedule system		List upcoming diagnostic and radiology tests in the following format: procedure name and scheduled date.
	Surgical records	Operating room system		Extract the following information from surgical records from the past 48 hours: procedure name, start time, end time, blood loss, and anesthesia method. If no records are available, state “No surgical records available.”
	Blood products	Blood bank system		Extract the following information on blood product orders within 48 hours: order number, blood type and Rh^a^ factor, order date, product name, quantity ordered/used, and transfusion date. If such information is unavailable, state “No blood product records available.”
	Wound records	Nursing information system		Extract the following wound assessment data: type, location, assessment date/time, and dimensions. If these data are unavailable, state “No wound assessment records available.”
	Consultation records	Medical documentation system		Summarize consultation findings and recommendations in Standard Mandarin. If such information is unavailable, state “No consultation records available.”
	Recommendations	Blank field		Generate comprehensive nursing care recommendations based on the provided data. Present recommendations as bullet points in only Standard Mandarin. Include information on priority interventions, monitoring requirements, preventive measures, and areas for special attention.
**Others**				
	Others	Blank field for manual data entry		N/A^b^

^a^Rh factor: Rhesus factor.

^b^N/A: not applicable.

### Interactions Between the NIS and the LLM in the Designed Workflow for Nursing Handover Documentation

Google Gemini 1.5 Pro offers an API that provides access to its LLM, which was integrated into the in-house NIS to automatically generate nursing handover documents.

**Figure 2:**
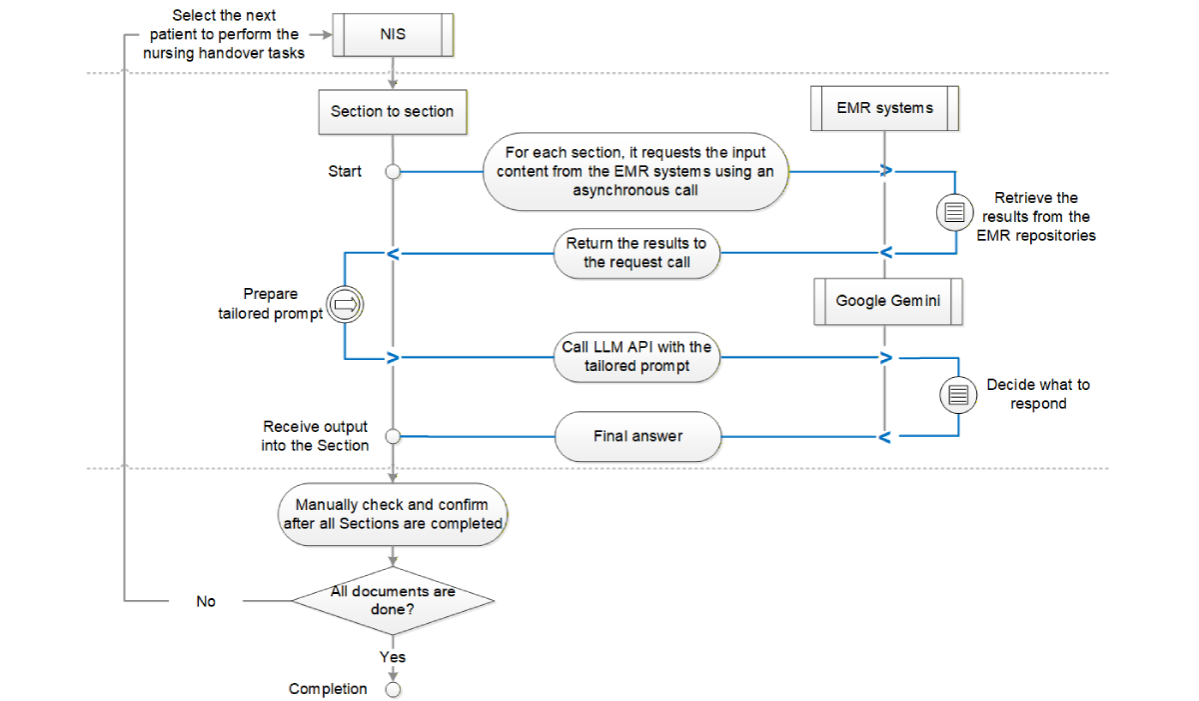
Integrated workflow for the large language model (LLM)–integrated nursing information system (NIS). API: application programming interface; EMR: electronic medical record.

Figure 2 illustrates the integrated workflow of the LLM-integrated NIS. For each section of the nursing handover document, the NIS sends a request to the relevant EMR system to retrieve patient data through asynchronous calls. The retrieved data and the corresponding tailored prompt are then used as input to call the LLM API. Upon receiving the LLM response, the NIS organizes the content and formats it according to the appropriate style for each section. The final output generated by the NIS is a structured and comprehensive handover document that can be manually reviewed and validated by nursing staff.

### Log Data Collection and Analysis of the Nursing Handover Document in the NIS

In this study, log data from the LLM-integrated NIS, implemented in October 2024, were used to conduct a preliminary pre-post comparison of nursing handover document completion times. Handover document completion time was defined as the interval from when a nurse selected a patient by clicking the handover button, through manual entry before GenAI integration or automatic draft generation after integration, to final confirmation, when the nurse completed review and revision and clicked the “Confirm” button for that patient.

To support subsequent analyses and system optimization, step-level log data were collected across the full handover documentation workflow. We retrospectively extracted NIS logs from September 2024 (preintegration) and from October-December 2024 (postintegration) for the 3 TMU-managed hospitals. To reduce the influence of extreme values, log data with handover document completion times exceeding 20 minutes were excluded from the analyses. In addition, we logged both the automatically GenAI-generated handover draft and the final nurse-confirmed version to preliminarily quantify the document revision rate. As nurse identifiers were unavailable, we used hospital × shift as the clustering unit and applied robust (sandwich) SEs to account for within-cluster correlation.

### Ethical Considerations

This retrospective study of deidentified NIS logs was approved by the TMU-Joint Institutional Review Board (TMU-JIRB; approval number N202506094). All data were deidentified to protect participant privacy and confidentiality, and no personally identifiable information was collected or disclosed. The TMU-JIRB waived the requirement for informed consent. All images included in the manuscript were reviewed to ensure that no individual participants or users could be identified. The hospitals also conduct routine qualitative and quantitative medical record audits, providing organizational accountability and quality assurance for AI-assisted documentation.

## Results

### Descriptive Statistics of Nursing-Related Factors for the Examined Hospitals

From September 1 to December 31, 2024, we collected NIS log data from the 3 hospitals during daily nursing handovers across 3 shifts (day, night, and overnight). At TMU, nursing handover documents must be prepared for most inpatients; however, certain patient categories are exempt from this requirement. These categories include patients undergoing postoperative observation, day hospitalization, and 1-day chemotherapy at TMUH; nursing home residents and day-care patients at WFH; and day-care, psychiatric ward, nursing home, and postpartum home care patients at SHH.

During the 4-month study period, the number of nurses working across the 3 daily shifts ranged from 501 to 524 at TMUH, 468 to 489 at WFH, and 862 to 892 at SHH. Monthly inpatient case volumes ranged from 7477 to 7733 at TMUH, 5406 to 5977 at WFH, and 8872 to 9814 at SHH. The mean monthly length of stay was 4.7-4.9 days at TMUH, 4.7-5.1 days at WFH, and 4.9-5.0 days at SHH. Across all shifts, total monthly shift counts ranged from 35,527 to 37,892 at TMUH, 27,500 to 30,835 at WFH, and 42,535 to 44,945 at SHH. Multimedia Appendix 1 summarizes descriptive statistics for the 3 hospitals included in this study.

### Comparison of the Original and GenAI-Assisted Nursing Handover Documentation Functions in the NIS

**Figure 3:**
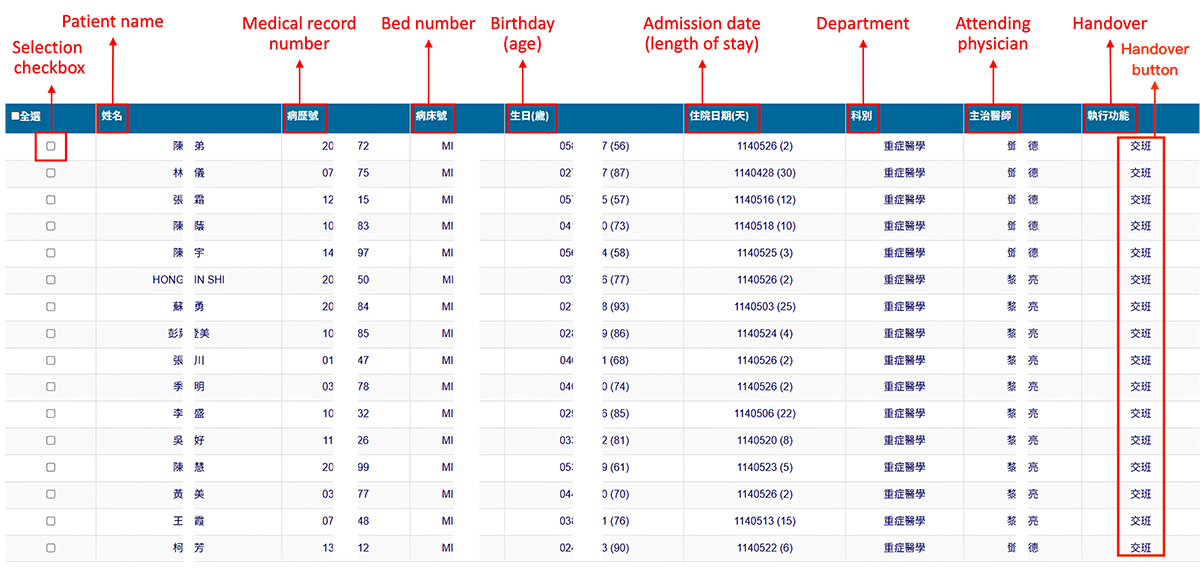
Screenshot of the patient list interface used by nurses to perform handover tasks in the nursing information system.

In accordance with the workflow shown in Figure 1, the LLM-integrated NIS initially displays a table listing patients, enabling nurses to generate handover documents sequentially by clicking a selection checkbox (Figure 3). Each row presents key patient attributes, including the patient’s name, medical record number, bed number, birth date and age, admission date and length of stay, department, and attending physician. The table also includes a handover button to initiate the nursing handover process.

**Figure 4:**
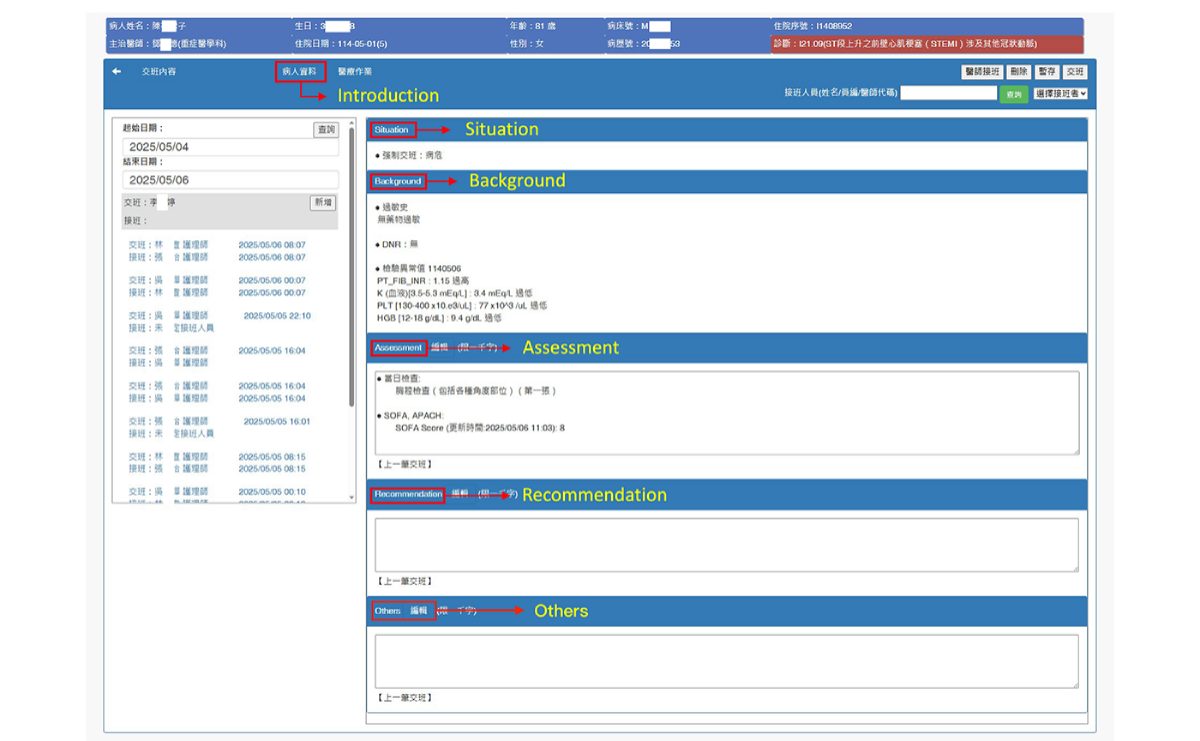
Screenshot of the Identify, Situation, Background, Assessment, Recommendation interface used for nursing handover tasks in the original nursing information system.

Figure 4 depicts the nursing handover documentation interface in the original NIS, displayed when a nurse selects a patient and initiates the handover process. Nurses complete 5 handover sections: Introduction (auto-populated; no manual entry required), Situation, Background, Assessment, and Recommendation and Other information.

**Figure 5:**
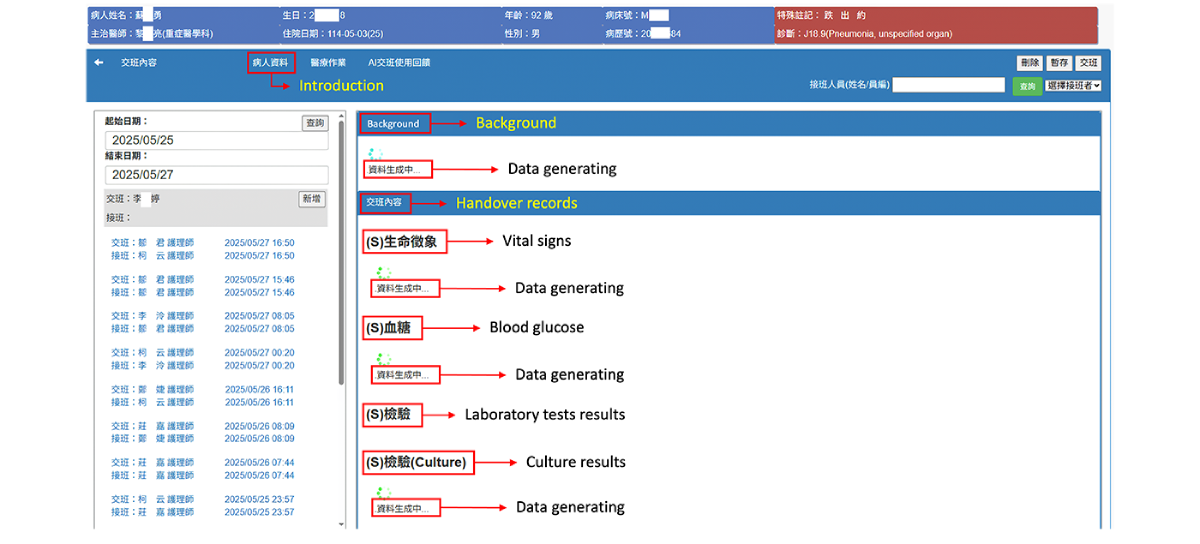
Screenshot of the revised nursing handover document in the large language model–integrated nursing information system.

**Figure 6:**
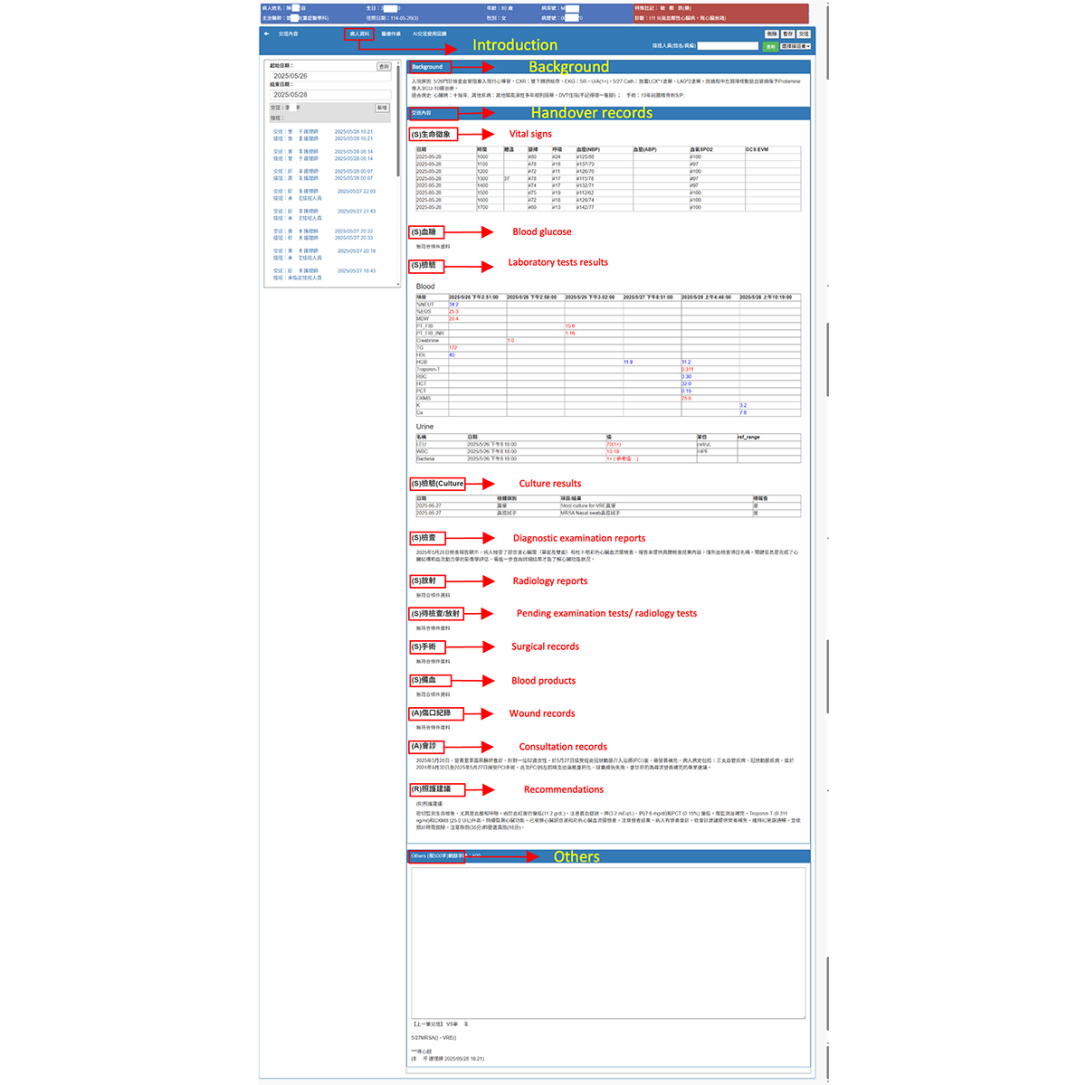
Screenshot of a complete nursing handover document automatically generated by the large language model–integrated nursing information system.

Figure 5 depicts the revised nursing handover document in the LLM-integrated NIS. In contrast to the original NIS, selecting a patient for handover triggers an API call to the LLM. After the response is returned, the LLM-integrated NIS automatically populates draft content for each handover section. Nurses then review, revise, and confirm the draft. Once finalized, the nurse clicks “Confirm” to submit the handover document for that patient (Figure 6).

### Analysis of NIS Log Data for Pre-LLM Integration

Table 4 presents detailed NIS log data from September 2024 for TMUH, WFH, and SHH, including the number of shifts and the mean (SD) of handover document completion time per patient for each shift. Before LLM integration, nurses often had to pause documentation to gather additional patient information or respond to unplanned clinical events (eg, bedside care, family inquiries, or urgent clinical duties). Accordingly, the NIS included a draft-save function that allowed handover content to be temporarily stored and completed later.

A total of 110,457 shift-level handover logs were analyzed (TMUH: 37,087; WFH: 30,835; and SHH: 42,535), representing large-scale real-world clinical operations. Across hospitals and shifts, the mean handover document completion time per patient ranged from 3.45 (SD 3.82) to 4.32 (SD 4.48) minutes. At TMUH, mean completion times were 4.10 (SD 4.55) minutes (day shift), 4.32 (SD 4.48) minutes (night shift), and 3.45 (SD 3.82) minutes (overnight shift). At WFH, the corresponding values were 4.26 (SD 5.08), 3.70 (SD 4.47), and 4.10 (SD 5.29) minutes, respectively, whereas at SHH they were 3.67 (SD 4.31), 4.09 (SD 4.25), and 3.81 (SD 4.23) minutes, respectively.

Although only 1 month of preintegration log data were available, these results provide a preliminary baseline of nursing handover documentation performance against which the impact of subsequent GenAI-assisted workflows can be quantitatively evaluated.

**Table 4:** Shift counts and mean document completion time, stratified by hospital and shift type before large language model integration (September 2024).

Hospital and shift		September 24	
		Number of shifts, n	Document completion time (minutes), mean (SD)
**Taipei Medical University Hospital** **(n=37,087)**			
	Day	12,418	4.10 (4.55)
	Night	12,203	4.32 (4.48)
	Overnight	12,466	3.45 (3.82)
**Wan Fang Hospital** **(n=30,835)**			
	Day	9379	4.26 (5.08)
	Night	10,547	3.70 (4.47)
	Overnight	10,909	4.10 (5.29)
**Shuang Ho Hospital** **(n=42,535)**			
	Day	13,868	3.67 (4.31)
	Night	14,466	4.09 (4.25)
	Overnight	14,201	3.81 (4.23)

### Analysis of NIS Log Data for LLM Responses

Table 5 summarizes shift-level log data from October to December 2024 for TMUH, WFH, and SHH, including the number of shifts, mean GenAI document generation time per patient, mean nurse manual (review-revision-confirmation) time per patient, and mean overall document completion time per patient. SHH had the highest shift volume (43,473 in October to 44,945 in December) and the largest monthly patient volume (8872-8989; Multimedia Appendix 1), with mean overall completion times ranging from 1.74 (SD 2.5) to 2.11 (SD 2.82) minutes. WFH recorded the lowest shift volume (27,500-28,730) and the shortest mean overall completion times (mean 1.17, SD 1.86 minutes to mean 1.78, SD 2.53 minutes) while managing 5746-5977 patients per month (Multimedia Appendix 1). TMUH showed intermediate shift volume (35,890-37,892) and patient volume (7477-7733; Multimedia Appendix 1), with mean overall completion times of 2.13 (SD 2.5) to 2.54 (SD 2.82) minutes.

We further conducted analyses using joint Wald tests to assess whether hospital, shift, and month were associated with statistically significant differences. As individual nurse identifiers were not available in the log data, clustering at the nurse level was not feasible. Therefore, we defined the clustering unit as hospital × shift, and the corresponding results are presented in Table 6.

In Table 6, joint Wald tests indicated that all 3 outcomes (GenAI time, manual time, and document completion time) differed significantly across hospitals and shifts (hospital: Wald *χ*^2^_2_=52.85, *P*<.001; shift: Wald *χ*^2^_2_=10.39, *P*=.006). By contrast, the effect of month was not statistically significant (Wald *χ*^2^_2_=5.74, *P*=.057).

Moreover, we used generalized estimating equations (GEEs) to account for within-cluster correlation arising from the clustered and longitudinal structure of the data. Separate GEE models were fitted for GenAI time, manual time, and document completion time. Each model included month, shift, and hospital as categorical covariates and used an exchangeable working correlation structure with robust SEs; thus, estimates represent population-averaged (system-level) effects rather than individual-level effects. Table 7 reports the population-averaged GEE results for GenAI time, manual time, and document completion time (β and SE). Relative to SHH, TMUH had longer times across all outcomes (GenAI time: β=3.05; manual time: β=20.35; document completion time: β=23.40; all *P*<.001), whereas WFH had shorter times (GenAI time: β=−3.47; manual time: β=−23.16; document completion time: β=−26.64; all *P*<.001). December (vs October) was associated with small increases across outcomes (*P*=.02), whereas November (vs October; *P*=.41) and shift (*P*=.44) were not statistically significant.

**Table 5:** Monthly shift-level patient volume and mean per-patient generative artificial intelligence, manual, and overall completion times across 3 Taipei Medical University hospitals (October-December 2024).

Hospital and shift			October 24									November 24									December 24						
			Number of shifts, n		Generative artificial intelligence time (seconds)^a^, mean (SD)		Manual time (minutes)^b^, mean (SD)		Document completion time (minutes)^c^, mean (SD)		Number of shifts, n			Generative artificial intelligence time (seconds), mean (SD)		Manual time (minutes), mean (SD)		Document completion time (minutes), mean (SD)		Number of shifts, n			Generative artificial intelligence time (seconds), mean (SD)		Manual time (minutes), mean (SD)		Document completion time (minutes), mean (SD)
**Taipei Medical University Hospital^d^**																											
	Day	12,043		17.0 (20.0)		1.89 (2.23)		2.17 (2.56)		11,882			16.70 (19.60)		1.85 (2.18)		2.13 (2.50)		12,510			17.00 (19.60)		1.88 (2.17)		2.17 (2.50)	
	Night	11,928		19.6 (22.1)		2.18 (2.45)		2.50 (2.82)		11,900			18.70 (22.10)		2.08 (2.45)		2.40 (2.82)		12,740			19.80 (22.10)		2.20 (2.45)		2.54 (2.82)	
	Overnight	11,918		19.0 (21.6)		2.11 (2.40)		2.43 (2.76)		11,745			19.00 (21.60)		2.11 (2.39)		2.42 (2.75)		12,642			18.60 (20.80)		2.07 (2.31)		2.38 (2.66)	
**Wan Fang Hospital^e^**																											
	Day	8291		12.1 (17.9)		1.34 (1.99)		1.54 (2.29)		8620			13.20 (19.50)		1.47 (2.17)		1.68 (2.50)		8905			13.90 (19.80)		1.55 (2.20)		1.78 (2.53)	
	Night	9612		12.5 (18.7)		1.39 (2.08)		1.60 (2.39)		10,035			12.50 (18.70)		1.38 (2.07)		1.59 (2.39)		10,190			12.50 (18.60)		1.39 (2.06)		1.60 (2.37)	
	Overnight	9597		9.2 (14.6)		1.02 (1.62)		1.17 (1.86)		9438			10.10 (15.40)		1.12 (1.72)		1.28 (1.97)		9635			9.60 (15.10)		1.07 (1.68)		1.23 (1.93)	
**Shuang Ho Hospital^f^**																											
	Day	13,103		14.8 (20.2)		1.65 (2.24)		1.89 (2.58)		13,931			15.40 (20.70)		1.71 (2.30)		1.97 (2.65)		13,875			15.30 (20.90)		1.70 (2.33)		1.96 (2.67)	
	Night	15,317		15.7 (21.3)		1.74 (2.36)		2.01 (2.72)		15,268			16.10 (21.50)		1.79 (2.39)		2.06 (2.75)		16,040			16.50 (22.00)		1.84 (2.45)		2.11 (2.82)	
	Overnight	15,053		13.7 (19.6)		1.52 (2.18)		1.74 (2.50)		15,337			14.10 (20.60)		1.57 (2.29)		1.80 (2.64)		15,030			15.60 (22.40)		1.74 (2.49)		2.00 (2.86)	

^a^Mean generative artificial intelligence document generation time per patient.

^b^Mean nurse manual (review-revision-confirmation) time per patient.

^c^Mean overall document completion time per patient.

^d^The total number of shifts for October, November, and December was 35,890, 35,527, and 37,892, respectively.

^e^The total number of shifts for October, November, and December was 27,500, 28,092, and 28,730, respectively.

^f^The total number of shifts for October, November, and December was 43,473, 44,536, and 44,945, respectively.

**Table 6:** Joint Wald tests for month, shift, and hospital effects on generative artificial intelligence time, manual time, and document completion time.

Outcome	Factor	Wald chi-square statistic (*df*)	*P* value
Generative artificial intelligence document generation time	Month	5.74 (2)	.06
Manual time^a^	Month	5.74 (2)	.06
Document completion time^b^	Month	5.74 (2)	.06
Generative artificial intelligence document generation time	Shift	10.39 (2)	.006
Manual time	Shift	10.39 (2)	.006
Document completion time	Shift	10.39 (2)	.006
Generative artificial intelligence document generation time	Hospital	52.85 (2)	<.001
Manual time	Hospital	52.85 (2)	<.001
Document completion time	Hospital	52.85 (2)	<.001

^a^Nurse manual (review-revision-confirmation) time.

^b^Overall document completion time per patient.

**Table 7:** Generalized estimating equation model coefficients for generative artificial intelligence document generation time, manual time, and document completion time.

Outcome	Factor	β (seconds)	95% CI	*P* value
Generative artificial intelligence document generation time	Intercept	15.09	14.26 to 15.92	<.001
Generative artificial intelligence document generation time	Hospital: TMUH^a^ vs SHH^b^	3.05	1.80 to 4.31	<.001
Generative artificial intelligence document generation time	Hospital: WFH^c^ vs SHH	–3.47	–4.72 to –2.23	<.001
Manual time^d^	Intercept	100.60	95.05 to 106.15	<.001
Manual time	Hospital: TMUH vs SHH	20.35	11.97 to 28.72	<.001
Manual time	Hospital: WFH vs SHH	–23.16	–31.45 to –14.88	<.001
Document completion time^e^	Intercept	115.69	109.31 to 122.07	<.001
Document completion time	Hospital: TMUH vs SHH	23.40	13.77 to 33.03	<.001
Document completion time	Hospital: WFH vs SHH	–26.64	–36.16 to –17.11	<.001
Generative artificial intelligence document generation time	Month: December vs October	.56	0.09 to 1.04	.02
Manual time	Month: December vs October	3.75	0.58 to 6.92	.02
Document completion time	Month: December vs October	4.31	0.66 to 7.95	.02
Generative artificial intelligence document generation time	Shift: night vs day	1.05	–0.24 to 2.34	.11
Manual time	Shift: night vs day	6.99	–1.61 to 15.60	.11
Document completion time	Shift: night vs day	8.04	–1.85 to 17.93	.11
Generative artificial intelligence document generation time	Month: November vs October	.16	–0.23 to 0.55	.41
Manual time	Month: November vs October	1.09	–1.50 to 3.69	.41
Document completion time	Month: November vs October	1.26	–1.73 to 4.24	.41
Generative artificial intelligence document generation time	Shift: overnight vs day	–.69	–2.45 to 1.07	.44
Manual time	Shift: overnight vs day	–4.59	–16.34 to 7.15	.44
Document completion time	Shift: overnight vs day	–5.28	–18.79 to 8.22	.44

^a^TMUH: Taipei Medical University Hospital.

^b^SHH: Shuang Ho Hospital.

^c^WFH: Wan Fang Hospital.

^d^Nurse manual (review-revision-confirmation) time.

^e^Overall document completion time per patient.

### Comparison of NIS Log Data Before and After LLM Integration

Using NIS logs from the preintegration period (September 2024) and the postintegration period (October-December 2024), we compared handover document completion time. Table 8 reports population-averaged GEE estimates for document completion time (seconds). Document completion time was significantly longer preintegration than postintegration (β=63.48 seconds; 95% CI 53.81-73.16; *P*<.001), and the overall period effect was significant (*χ*^2^_1_=165.24; *P*<.001). Relative to SHH, completion time was longer at TMUH (β=19.10 seconds; 95% CI 10.34-27.85; *P*<.001) and shorter at WFH (β=−25.67 seconds; 95% CI −34.42 to −16.92; *P*<.001). Shift effects were not statistically significant (night vs day, *P*=.15; overnight vs day, *P*=.33).

**Table 8:** Generalized estimating equation model coefficients for handover document completion time comparing pre- and postintegration periods.

Outcome	Factor	β (seconds)	95% CI	*P* value
Document completion time^a^	Intercept	118.01	112.21 to 123.81	<.001
Document completion time	Pre^b^ vs Post^c^	63.48	53.81 to 73.16	<.001
Document completion time	Taipei Medical University Hospital vs Shuang Ho Hospital	19.10	10.34 to 27.85	<.001
Document completion time	Wan Fang Hospital vs Shuang Ho Hospital	–25.67	–34.42 to –16.92	<.001
Document completion time	Night vs day	6.44	–2.35 to 15.22	.15
Document completion time	Overnight vs day	–5.98	–17.99 to 6.03	.33

^a^Overall document completion time per patient.

^b^Preintegration document completion time.

^c^Postintegration document completion time.

### Revision Rate of GenAI-Generated Handover Drafts

Using NIS logs from October to December 2024, we examined revisions of GenAI-generated handover drafts by hospital and shift. Table 9 presents, for each hospital-shift stratum, the number of patient-level handover documents in which nurses revised the GenAI-generated draft (number of documents with revisions), the total number of patient-level handover documents (number of documents), and the corresponding revision rate (number of documents with revisions/number of documents, %).

Revisions were common across hospitals and months. At TMUH, revision rates were higher during night (9267/12,740-9162/11,928, 72.74%-76.81%) and overnight shifts (9551/11,918-10,830/12,642, 80.14%-85.67%) than during day shifts (8343/12,510-8164/12,043, 66.69%-67.79%). At WFH, day-shift revision rates increased from nearly 50% (4215/8291, 50.84%) in October to over 60% (5334/8620-5562/8905, 61.88%-62.46%) in November-December, whereas night-shift revision rates decreased from nearly 79% (7684/9612, 79.94%) in October to nearly 60% (6000/10,035-6178/10,190, 59.79%-60.63%) thereafter; overnight-shift revision rates remained high (6960/9438-7804/9597, 73.74%-81.32%). At SHH, revision rates were relatively stable, with day shifts at 68.86%-74.16% (9554/13,875-9717/13,103) and night and overnight shifts at 73.95%-78.95% (11,861/16,040-11,884/15,053).

**Table 9:** Monthly patient-level handover document counts and revision rates of generative artificial intelligence–generated drafts by hospital and shift (October-December 2024).^a^

Hospital and shift		October 24			November 24				December 24		
		Number of documents with revisions^b^, n	Number of documents^c^, n	Revision rate^d^, %	Number of documents with revisions, n	Number of documents, n	Revision rate, %	Number of documents with revisions, n		Number of documents, n	Revision rate, %
**Taipei Medical University Hospital**											
	Day	8164	12,043	67.79	8001	11,882	67.34	8343		12,510	66.69
	Night	9162	11,928	76.81	9000	11,900	75.63	9267		12,740	72.74
	Overnight	9551	11,918	80.14	9744	11,745	82.96	10,830		12,642	85.67
**Wan Fang Hospital**											
	Day	4215	8291	50.84	5334	8620	61.88	5562		8905	62.46
	Night	7684	9612	79.94	6000	10,035	59.79	6178		10,190	60.63
	Overnight	7804	9597	81.32	6960	9438	73.74	7810		9635	81.06
**Shuang Ho Hospital**											
	Day	9717	13,103	74.16	9717	13,931	69.75	9554		13,875	68.86
	Night	11,886	15,317	77.60	11,886	15,268	77.85	11,861		16,040	73.95
	Overnight	11,884	15,053	78.95	11,884	15,337	77.49	11,759		15,030	78.24

^a^Each log entry corresponds to 1 handover document for a single patient within a shift.

^b^“Number of documents with revisions” denotes documents in which the generative artificial intelligence–generated draft was edited before confirmation.

^c^“Number of documents” denotes the total number of patient-level handover documents.

^d^The revision rate (%) was calculated as documents with revisions/number of documents × 100.

### Estimated Time Savings in Handover Documentation Before and After LLM Integration

Nurses at TMUH, WFH, and SHH estimated that they typically completed handover documentation for 5-7 patients per shift when using the original NIS. Based on the available preintegration logs (limited to 1 month), the average documentation completion time ranged from 3.45 (SD 3.82) to 4.32 (SD 4.48) minutes per patient across hospitals and shifts (Table 4). Postintegration logs from October to December 2024 (Table 5) further characterized workflow performance by decomposing document completion time into GenAI generation time and nurse review-revision-confirmation time; the average overall completion time per patient ranged from 1.17 (SD 1.86) to 2.54 (SD 2.82) minutes. Taken together, these figures suggest a potential absolute reduction of approximately 0.91-3.15 minutes per patient ([3.45-2.54]/3.45-[4.32-1.17]/4.32, 26%-73% relative reduction). Moreover, using monthly patient volumes and the observed reduction in per-patient completion time, the LLM-integrated workflow corresponds to estimated savings of approximately 470-980 hours per month across the 3 hospitals (Table 10). Full-time equivalent (FTE) is a standard unit used to express workload or staffing capacity as the work time of 1 full-time worker over a specified period. FTE equivalents were calculated assuming 160 nursing work hours per FTE-month. At TMUH, the projected reduction was approximately 113-282 hours per month (equivalent to 0.7-1.8 FTE/month, assuming 160 working hours per FTE). At WFH, the estimated savings were approximately 160-314 hours per month (1.0-2.0 FTE/month). At SHH, which had the highest patient volume, the estimated savings were approximately 198-387 hours per month (1.2-2.4 FTE/month).

**Table 10:** Estimated monthly nursing time savings after large language model integration, by hospital (hours and full-time equivalents).^a^

Hospital	October 24, hours (full-time equivalent)^b^	November 24, hours (full-time equivalent)^b^	December 24, hours (full-time equivalent)^b^	Average, hours (full-time equivalent)^b^
Taipei Medical University Hospital	113-273 (0.71-1.71)	115-276 (0.72-1.72)	117-282 (0.73-1.76)	115-277 (0.72-1.73)
Wan Fang Hospital	163-307 (1.02-1.92)	166-314 (1.04-1.96)	160-302 (1.00-1.89)	163-308 (1.02-1.92)
Shuang Ho Hospital	198-381 (1.24-2.38)	203-391 (1.27-2.44)	201-387 (1.25-2.42)	201-386 (1.25-2.41)
Total	474-962 (2.96-6.01)	484-981 (3.02-6.13)	478-970 (2.99-6.07)	479-971 (2.99-6.07)

^a^Hours saved/month = patients × (minutes saved/patient)/60. Preintegration times (September 2024 only; Table 3) were 3.45-4.32 minutes/patient. Postintegration times (October-December 2024; Table 4) were as follows: Taipei Medical University Hospital, 2.13-2.54 minutes/patient; Wan Fang Hospital, 1.17-1.78 minutes/patient; and Shuang Ho Hospital 1.74-2.11 minutes/patient. Bounds were computed as pre-min – post-max and pre-max – post-min.

^b^Values are listed as ranges.

### Cost-Effectiveness of GenAI-Assisted Handover Documentation

Preliminary GenAI token cost-effectiveness was evaluated by comparing GenAI operating expenditures with estimated nursing time savings (Table 11). Monthly patient volume was used to approximate documentation time saved, and nursing time was valued at US $10 per hour to estimate the equivalent labor value (opportunity cost) of time freed for clinical work (ie, this does not represent direct budget savings unless staffing levels or overtime are reduced). On a monthly basis, allocated GenAI operating costs were approximately US $493-US $986 per hospital, corresponding to an estimated 113-391 hours saved per hospital per month. The resulting monthly net labor value ranged from US $322 to US $2100 at TMUH, US $970 to US $2595 at WFH, and US $1024 to US $3082 at SHH. Aggregated across hospitals, the estimated time savings were 474-981 hours per month, equivalent to US $4740-US $9810 in labor value. After subtracting monthly GenAI operating expenditures (US $1871-US $2464), the implied net labor value was approximately US $2276-US $7939 per month. Over the 3-month period, the total GenAI cost was US $6396.97, with an estimated 1436-2913 hours saved, yielding an overall net labor value of US $7963-US $22,733.

**Table 11:** Estimated monthly generative artificial intelligence operating costs, hours saved, equivalent labor value, and net labor value by hospital (October-December 2024).

Hospital and measure		October 24	November 24	December 24	Overall (October to December)
**Taipei Medical University Hospital**					
	Allocated generative artificial intelligence cost, US $^a^	630.18	688.92	848.02	2167.11
	Time saved (hours)^b^, range	113-273	115-276	117-282	345-831
	Equivalent labor value (US $10/hour), range	1130-2730	1150-2760	1170-2820	3450-8310
	Net labor value^c^ (US $), range	499.82-2099.82	461.08-2071.08	321.98-1971.98	1282.89-6142.89
**Wan Fang Hospital**					
	Allocated generative artificial intelligence cost, US $	493.14	544.74	630.12	1667.99
	Time saved (hours), range	163-307	166-314	160-302	489-923
	Equivalent labor value (US $10/hour), range	1630-3070	1660-3140	1600-3020	4890-9230
	Net labor value (US $), range	1136.86-2576.86	1115.26-2595.26	969.88-2389.88	3222.01-7562.01
**Shuang Ho Hospital**					
	Allocated generative artificial intelligence cost, US $	747.75	828.36	985.75	2561.87
	Time saved (hours), range	198-381	203-391	201-387	602-1159
	Equivalent labor value (US $10/hour), range	1980-3810	2030-3910	2010-3870	6020-11,590
	Net labor value (US $), range	1232.25-3062.25	1201.64-3081.64	1024.25-2884.25	3458.13-9028.13
**All**					
	Allocated generative artificial intelligence cost, US $	1871.07	2062.02	2463.89	6396.97
	Time saved (hours), range	474-961	484-981	478-971	1436-2913
	Equivalent labor value (US $10/hour), range	4740-9610	4840-9810	4780-9710	14,360-29,130
	Net labor value (US $), range	2868.93-7738.93	2777.98-7747.98	2316.11-7246.11	7963.03-22,733.03

^a^Allocated generative artificial intelligence costs were distributed according to each hospital’s monthly patient-volume share.

^b^Time saved was estimated from monthly patient volumes and the reduction in per-patient handover document completion time.

^c^Net labor value was calculated as equivalent labor value − allocated generative artificial intelligence cost.

## Discussion

### Principal Findings

In this study, we integrated Google Gemini 1.5 Pro into the in-house NIS of TMU Health to improve the efficiency of nursing handover documentation across 3 hospitals in Taiwan. In the LLM-integrated workflow, a nurse initiates handover by selecting a patient, after which the LLM-integrated NIS retrieves relevant patient data and submits section-specific, tailored prompts to the LLM. The LLM generates draft content for each handover section, and the compiled document is returned to the LLM-integrated NIS for nurse review, revision, and finalization. This integration streamlines handover documentation by reducing manual data entry and supporting a more efficient routine workflow.

The LLM-integrated NIS retains the functionalities of the original in-house NIS, thereby enabling seamless incorporation of the LLM into the existing nursing handover documentation workflow. Nurses remain familiar with the system interfaces, obviating the need for adaptation and minimizing challenges related to digital skills training and system usability. Furthermore, nurses can readily interact with the LLM-integrated NIS and access the generated content, ensuring that the system does not interrupt workflow [42,43]. Consequently, nurses can maintain their focus on patient care while benefiting from the time-saving features of the LLM, ultimately improving both clinical and operational outcomes.

Across 3 TMU Health hospitals, postintegration NIS logs showed shorter per-patient handover documentation completion times than the limited 1-month preintegration baseline, corresponding to an estimated reduction of 0.91-3.15 minutes per patient ([3.45-2.54]/3.45-[4.32-1.17]/4.32, 26%-73%). When scaled by monthly patient volumes (Table 10), this reduction translated to approximately 470-980 hours saved per month across hospitals (approximately 0.7-1.8 FTE/month, assuming 160 working hours per FTE). A preliminary token cost evaluation suggested that GenAI operating expenditures (US $493-US $986 per hospital per month) were modest relative to the estimated labor or opportunity value of nursing time freed for clinical work, yielding a positive net labor value across October-December 2024 (Table 11). Overall, these findings suggest that GenAI-assisted handover documentation may reduce documentation burden and return clinically meaningful nursing time that could be redirected to direct patient care, which is particularly relevant in the context of nursing workforce shortages.

### Safety Considerations and Hallucination Mitigation

Hallucinations are a well-recognized limitation of GenAI, particularly in clinical documentation tasks in which factual accuracy is critical [44-46]. During initial development and testing of the LLM-integrated NIS, we observed occasional hallucinations when the LLM was used to extract and structure vital signs from NIS inputs; for example, the model sometimes generated an average body temperature that was not explicitly documented in the source record. To mitigate hallucination risk, we implemented multiple safeguards. First, we iteratively refined prompts to restrict the LLM to reformatting and summarizing only the provided input and to prohibit the introduction of new values or inferences. Second, we required nurse review, correction, and confirmation before finalizing each handover note, thereby adding an additional verification layer. Despite these measures, we considered the residual risk of LLM-generated structured vital-sign outputs to be clinically unacceptable. Accordingly, we discontinued LLM-based structuring of vital signs and instead directly displayed the patient’s vital signs in the NIS. By contrast, the LLM appeared more reliable for extracting and synthesizing narrative information (eg, abnormal findings and clinically relevant descriptions) from radiology and examination reports, in which the task emphasized highlighting salient text rather than generating or imputing numeric values.

### Limitations

First, only 1 month of preintegration log data were available, limiting the robustness of the baseline for manual handover documentation and constraining pre-post comparisons. Accordingly, the preintegration results should be interpreted as a preliminary benchmark for estimating the potential impact of subsequent GenAI-assisted workflows. Second, the NIS log schema was not sufficiently granular to support several secondary analyses. Specifically, we could not quantify technical reliability indicators (eg, connection failures, API time-outs, error returns, failed generations, or regeneration attempts), nor could we stratify performance by patient complexity (eg, intensive care unit vs general ward) or clinical acuity proxies such as length of stay. Consequently, the reported time and cost estimates reflect system-level averages and may mask heterogeneity in performance for complex cases or under high-load conditions. Third, we did not conduct a formal natural language processing evaluation of generated text quality using standard metrics or a gold-standard reference set [47,48]. Although nursing professionals reviewed and verified the LLM-generated handover content as part of routine workflow, the absence of quantitative natural language processing evaluation limits comparability with prior summarization studies and precludes objective assessment of section-level content fidelity. Fourth, although we quantified the revision rate of GenAI-generated drafts, we did not classify edit types (eg, corrections vs clarifications vs additions) or examine the reasons underlying nurse modifications. Therefore, we could not determine which content areas most frequently required changes or whether revisions primarily reflected model limitations, workflow preferences, or patient- and context-specific factors. Fifth, we did not conduct a formal usability or user experience evaluation. Specifically, we did not administer validated instruments (eg, the System Usability Scale or Technology Acceptance Model) or conduct structured interviews to assess nurses’ perceived workload, trust in GenAI outputs, or overall satisfaction, limiting conclusions about user acceptability beyond log-based performance metrics.

### Future Directions

Our preliminary findings suggest that LLMs can support rapid, automated generation of nursing handover documents; however, nurse review and revision remain necessary before finalization. First, future work will expand the NIS logging framework to capture reliability and workflow metrics (eg, time-outs, error codes, retries, regeneration events, and editing behaviors) and enable more granular performance evaluation, including downstream operational outcomes such as nursing overtime hours. Second, we will compare LLM-generated drafts with nurse-finalized handovers to characterize error patterns, quantify revision types, and inform iterative prompt and model refinement. These analyses will be guided by predefined quality indicators and evaluation frameworks (eg, quantitative and qualitative ISBAR-based assessments) in future studies. Collectively, these data will support improvements in factual accuracy and the development of additional safeguards, including rule-based checks and retrieval-augmented generation strategies [49]. Third, to evaluate usability and acceptance, we will incorporate standardized instruments and structured qualitative feedback to assess nurse-perceived usability, trust, cognitive load, and workflow impact alongside log-derived efficiency outcomes. Fourth, to improve transferability, we will evaluate interoperability in settings with different EMR architectures and develop a configurable prompt-and-template layer (eg, leveraging HL7/FHIR [Health Level 7/Fast Healthcare Interoperability Resources] where available) to reduce site-specific customization. Specifically, we aim to reduce institution-specific effort related to data mapping; integration with source systems (eg, laboratory information system/radiology information system/examination information system); authentication and authorization; local handover templates and documentation policies; and network or operational configurations. In parallel, we will preserve and further validate transferable components, including ISBAR-aligned section prompts, the human-in-the-loop workflow (draft generation with nurse review-revision-confirmation), safety safeguards (eg, prompt constraints and use of source-of-truth structured data), and a log-based evaluation framework for efficiency, revision behavior, and reliability metrics. Finally, we will assess generalizability to other clinical documentation workflows, including automated note drafting and radiology report generation.

### Conclusions

In this study, we integrated an LLM into an existing NIS to generate nursing handover documents in routine practice without modifying the user interface or disrupting established workflows. Across 3 hospitals within TMU Health, GenAI-assisted handover documentation was associated with reduced per-patient documentation completion time in real-world use. A preliminary cost-effectiveness analysis suggested that GenAI operating expenditures were modest relative to the estimated labor value of nursing time freed for clinical work from October to December 2024. To mitigate hallucination risk, particularly for structured numeric data, we constrained prompts and embedded nurse review, editing, and confirmation within the workflow. Overall, these findings support the operational feasibility of integrating GenAI into hospital documentation workflows while retaining human verification. Future work will expand NIS logging to quantify reliability and editing metrics, compare LLM-generated drafts with nurse-finalized documents to identify error patterns and inform prompt and model refinement, and evaluate interoperability and generalizability to other clinical documentation tasks.

## Appendix

Multimedia Appendix 1 Monthly patient volume, nurse staff levels, shift counts, and mean length of stay, stratified by hospital and shift type (September-December 2024).

## Abbreviations

AI: artificial intelligence
API: application programming interface
EMR: electronic medical record
FHIR: Fast Healthcare Interoperability Resources
FTE: full-time equivalent
GEE: generalized estimating equation
GenAI: generative artificial intelligence
HIPAA: Health Insurance Portability and Accountability Act
HL7: Health Level 7
ISBAR: Identify, Situation, Background, Assessment, Recommendation
JIRB: Joint Institutional Review Board
LLM: large language model
NIS: nursing information system
RIS: radiology information system
SHH: Shuang Ho Hospital
TMU: Taipei Medical University
TMUH: Taipei Medical University Hospital
WFH: Wan Fang Hospital
WHO: World Health Organization

## Data Availability

The data are available upon reasonable request from the corresponding author (YSL) and with TMU-JIRB approval; they are not publicly available due to ethical and privacy restrictions.
