# Supplementary material for: Integrating a Large Language Model to Streamline Nursing Handover Documentation Across Multiple Hospitals in Taiwan: Development and Implementation Study
Source: J Med Internet Res. 2026 Mar 12;28:e81604. doi: 10.2196/81604 (PMC13022550; doi:10.2196/81604)
Supplement: Multimedia Appendix 1 [file jmir_v28i1e81604_app1.docx]

**Table 3.** Monthly patient volume, nurse staff levels, shift counts, and mean length of stay, stratified by hospital and shift type (September-December 2024).

| H | Shift | September 24 | | | | | October 24 | | | | | November 24 | | | | | December 24 | | | |
| --- | --- | --- | --- | --- | --- | --- | --- | --- | --- | --- | --- | --- | --- | --- | --- | --- | --- | --- | --- | --- |
|  |  | Number of patients | Number of nurses | Mean LOS | Number of shifts | Number of patients | | Number of nurses | Mean LOS | Number of shifts | Number of patients | | Number of nurses | Mean LOS | Number of shifts | Number of patients | | Number of nurses | Mean LOS | Number of shifts |
| TMUH | D | 2598 | 213 | 4.8 | 12,418 | 2509 | | 212 | 4.8 | 12,043 | 2528 | | 219 | 4.7 | 11,882 | 2553 | | 199 | 4.9 | 12,510 |
|  | N | 2553 | 181 |  | 12,203 | 2485 | | 175 |  | 11,928 | 2532 | | 169 |  | 11,900 | 2600 | | 170 |  | 12,740 |
|  | O | 2608 | 130 |  | 12,466 | 2483 | | 131 |  | 11,918 | 2499 | | 126 |  | 11,745 | 2580 | | 132 |  | 12,642 |
|  | Total | 7618 | 524 |  | 37,087 | 7477 | | 518 | -- | 35,889 | 7559 | | 514 | -- | 35,527 | 7733 | | 501 | -- | 37,892 |
| WFH | D | 1830 | 220 | 5.1 | 9379 | 1764 | | 234 | 4.7 | 8291 | 1834 | | 214 | 4.7 | 8620 | 1781 | | 207 | 5.0 | 8905 |
|  | N | 1746 | 144 |  | 10,547 | 2045 | | 145 |  | 9612 | 2135 | | 147 |  | 10,035 | 2038 | | 149 |  | 10,190 |
|  | O | 1830 | 119 |  | 10,909 | 2042 | | 110 |  | 9597 | 2008 | | 107 |  | 9438 | 1927 | | 118 |  | 9635 |
|  | Total | 5406 | 483 | -- | 30,835 | 5851 | | 489 | -- | 27,500 | 5977 | | 468 | -- | 28,093 | 5746 | | 474 | -- | 28,730 |
| SHH | D | 3300 | 368 | 4.9 | 13,868 | 2674 | | 386 | 4.9 | 13,103 | 2843 | | 377 | 4.9 | 13,931 | 2775 | | 363 | 5.0 | 13,875 |
|  | N | 3212 | 269 |  | 14,466 | 3126 | | 270 |  | 15,317 | 3116 | | 289 |  | 15,268 | 3208 | | 265 |  | 16,040 |
|  | O | 3302 | 225 |  | 14,201 | 3072 | | 221 |  | 15,053 | 3130 | | 226 |  | 15,337 | 3006 | | 238 |  | 15,030 |
|  | Total | 9814 | 862 | -- | 42,535 | 8872 | | 877 | -- | 43,473 | 9089 | | 892 | -- | 44,536 | 8989 | | 866 | -- | 44,945 |
| Abbreviations: H, Hospital; TMUH, Taipei Medical University Hospital; WFH, Wan Fang Hospital; SHH, Shuang Ho Hospital; D, Day; N, Night; O, Overnight; Mean LOS, Mean length of stay | | | | | | | | | | | | | | | | | | | | |
